# Analysis of blood degradation products and ferritin in the cerebrospinal fluid of dogs with acute thoracolumbar intervertebral disk extrusion, a prospective pilot study

**DOI:** 10.1186/s12917-019-1878-9

**Published:** 2019-05-14

**Authors:** Sophie Bittermann, Christof Schild, Eliane Marti, Jelena Mirkovitch, Daniela Schweizer, Diana Henke

**Affiliations:** 1 Division of Neurological Sciences, Division of Clinical Veterinary Neurology, Department of Clinical Veterinary Medicine, Vetsuisse Faculty, University of Bern, Laenggassstrasse 128, 3012 Bern, Switzerland; 2 Institute of Clinical Chemistry, Inselspital, Bern University Hospital and University of Bern, INO-F3010, Bern, Switzerland; 3 Department of Clinical Research and Veterinary Public Health, Vetsuisse Faculty, University of Bern, Laenggassstrasse 128, 3012 Bern, Switzerland; 4 Division of Neurological Sciences, Division of Veterinary Radiology, Department of Clinical Veterinary Medicine, Vetsuisse Faculty, University of Bern, Laenggassstrasse 128, 3012 Bern, Switzerland

**Keywords:** Spinal cord injury, Oxyhemoglobin, Bilirubin, Subarachnoidal hemorrhage

## Abstract

**Background:**

Hemorrhage in the spinal canal leads to further damage of the spinal cord influencing outcome in dogs with intervertebral disk (IVD) extrusion. The aim of the study was to evaluate blood degradation products and ferritin in the cerebrospinal fluid (CSF) of dogs with thoracolumbar IVD extrusion, and their association to clinical parameters and MRI findings.

**Results:**

In the CSF of dogs with IVD extrusion, both net oxyhemoglobin absorption (NOA) and net bilirubin absorption (NBA) were significantly higher compared to the control groups of dogs with steroid responsive meningitis arteritis (SRMA) and idiopathic epilepsy (IE) (*P <* 0.001), but NOA compared to the idiopathic epilepsy group contaminated artificially with blood (IEc) was not (*P =* 0.890). Ferritin concentration was significantly higher in dogs with IVD extrusion compared to dogs with IE (*P =* 0.034), but not to dogs with SRMA (*P =* 0.526). There was no association between NOA, NBA or ferritin concentration and severity or duration of clinical signs. In dogs with a higher ferritin concentration the outcome was better (*P =* 0.018). In dogs with evidence of hemorrhage on MRI, NOA and NBA were significantly higher (*P =* 0.016, *P =* 0.009), but not ferritin (*P =* 0.0628).

**Conclusion and clinical importance:**

Quantification of blood degradation products and ferritin in the CSF of dogs to assess subarachnoidal hemorrhage is feasible; however, larger case numbers are needed to evaluate the relevance of NBA and ferritin as prognostic indicators.

## Background

In paraplegic dogs with thoracolumbar intervertebral disk (IVD) extrusion, the presence or absence of nociception is considered the most important indicator to determine the prognosis [1, 2], and generally correlates with histopathological findings [3]. However, the outcome of surgically treated paraplegic dogs with absent nociception varies widely [2, 4–6]. is highlights the fact that other factors beyond this contribute to recovery or lack thereof. Several other prognostic factors were previously studied including severity and duration of clinical signs, site of disk extrusion, specific magnetic resonance imaging (MRI) findings [7–9], and cerebrospinal fluid (CSF) characteristics [10–17].

After the primary mechanical insult on the spinal cord in IVD extrusion, secondary mechanisms of injury including decreased vascular perfusion followed by ischemia and perivascular edema, electrolyte imbalances, glutamatergic excitotoxicity, oxidative stress, inflammation, and apoptosis lead to further damage of the spinal cord parenchyma [18, 19]. Additionally, there is strong evidence that intramedullary and subdural hemorrhage has an important impact on the development of secondary injury and outcome [20, 21]. Therefore, monitoring hemorrhage in the subarachnoidal space would seem useful to assess spinal cord injury (SCI) and hence prognosis.

Following hemorrhage into the subarachnoidal space, rapid lysis of red blood cells occurs, thereby releasing oxyhemoglobin. The liberated oxyhemoglobin is gradually converted into bilirubin and free iron by macrophages and other cells of the leptomeninges [22]. The free iron is detoxified by ferritin [23, 24].

Detection of such products in CSF with spectrophotometry for net oxyhemoglobin absorption (NOA) and net bilirubin absorption (NBA) as well as detection of ferritin in the CSF is used in people with subarachnoidal hemorrhage (SAH) when computed tomography of the head is negative for hemorrhage [24, 27, 28]. Spectrophotometry for blood degradation products in the CSF of animals has been used experimentally in a model for human SAH [28, 29], but to our knowledge, never before to detect blood degradation products in the CSF in a naturally occurring disease. Neither have these techniques been used before to assess SCI.

The aim of the present pilot study was to investigate the utility of spectrophotometry and ferritin ELISA to quantify blood degradation products and ferritin in CSF of dogs with SCI following IVD extrusion. Further, we wanted to assess whether these degradation products and ferritin are correlated with neurological grade of affected dogs and their outcome, and whether they can be used as prognostic indicators. We hypothesized that quantification of blood degradation products and ferritin in the CSF could be a method to assess subdural hemorrhage and may serve as useful prognostic indicator because of its influence on secondary damage of the spinal cord [3, 20, 21, 25, 26]. Additionally, the correlation of blood degradation products and ferritin with MRI findings was evaluated.

## Results

### Clinical data

The study population consisted of 58 purebred dogs (33 different breeds), and 11 mixed-bred dogs. Pure breeds represented by more than three dogs were dachshunds (*n* = 10) and French bulldogs (*n* = 7). Forty-eight dogs were male (21 castrated), and 21 were female (13 spayed). The mean age of the dogs was 4.8 years (range, 6 months to 12.8 years). The median body weight of the dogs was 13.5 kg (range, 3.4 to 81.0 kg).

In dogs with IVD extrusion, the initial neurological condition was Grade I in 1 dog, Grade II in 12, Grade III in 9, Grade IV in 14, and Grade V in 3 dogs.

The median duration of the clinical signs until CSF tap was 2 days (range, 1 to 21 days), and the clinical signs were classified as acute in 20 dogs, subacute in 13 dogs, and chronic in 6 dogs, accordingly. Three of the chronically affected dogs displayed a worsening of clinical signs within the last 24 h.

The IVD extrusion site was at T11-T12 in three dogs, T12-T13 in 13 dogs, T13-L1 in 12 dogs, L1-L2 in one dog, L2-L3 in four dogs, L3-L4 in two dogs, L4-L5 in three dogs, and L5-L6 in one dog. Time of discharge or euthanasia was between 0 and 17 days (median 4). Thirty dogs had a good outcome and were ambulatory, one dog showed an improvement, but was not ambulatory at time of discharge. The remaining 8 dogs were euthanized immediately after imaging (*n* = 2 of each; neurological grade IV and V), after detoriation of neurological signs (*n* = 3; neurological grade II to III, grade II to V, and grade IV to V), and because of lack of improvement (*n* = 1, neurological grade IV).

### Magnetic resonance imaging

On MRI, hemorrhage was evident in 24 dogs, whereas in the 15 remaining dogs no signs of hemorrhage were visible.

### Serum bilirubin levels and cerebrospinal fluid analysis

Median values and ranges of serum bilirubin, CSF total protein, WBC count, NBA, NOA, and ferritin concentration, results of pandy testing and CSF cytology, and the classification according to Cruickshank et al. of the different groups are given in Table 1.

**Table 1:** Median values and ranges of serum bilirubin levels, cerebrospinal fluid analysis, spectrophotometric and ferritin measurements

	Intervertebral disc extrusion	Idiopathic epilepsy	Idiopathic epilepsy contaminated	Steroid-responsive meningitis-arteritis
	(*n* = 39)	(*n* = 21)	(n = 21)	(*n* = 9)
Routine CSF analysis				
WBC / μl	*			
median (range)	8 (1–14)	0.67 (0–5)		47.67 (66–1680)
TP (g/l)	*			
median (range)	0.73 (0.14–8.55)	0.12 (0.11–0.23)		0.25 (0.15–0.8)
Pandy (number)	*			
–	2	17		5
+	3	4		4
++				
+++	1			
Cytology (number)				
*mononuclear*	2			1
*neutrophilic*	1			8
CSF spectrophotometry				
NOA (AU)	**			
median (range)	0.073 (0–3.585)	0.002 (0–0.009)	0.064 (0.003–1.505)	0.004 (0–0.010)
NBA (AU)	**			
median (range)	0.016 (0–0.373)	0.001 (0–0.007)	0.001 (0–0.011)	0.002 (0.001–0.008)
ELISA CSF Ferritin (ng/ml)				
median (range)	41.28 (15.08–228.19)	33.90 (13.58–64.00)	36.73 (15.00–653.30)	31.11 (17.84–125.14)
Serum Bilirubin (μmol/l)				
median (range)	2 (0.7–6)	2 (0.7–6)		2 (1–4.2)
Classification of the result according to Cruickshank [27] Subarachnoidal hemorrhage:				
yes	26	0	1	1
no	6	21	13	8
not excluded	1	0	7	0

*performed in 6 dogs,** performed in 33 dogs, WBC, white blood cell count; TP, total protein; AU, absorption units; NOA, net oxyhemoglobin absorption; NBA net bilirubin absorption; CSF, cerebrospinal fluid

Spectrophotometric analysis of NOA and NBA has been performed in 33 of 39 dogs with IVD extrusion, and routine CSF analysis in 6 of these 33 dogs because of insufficient quantity of CSF.

### Statistical analysis

**Fig. 1:**
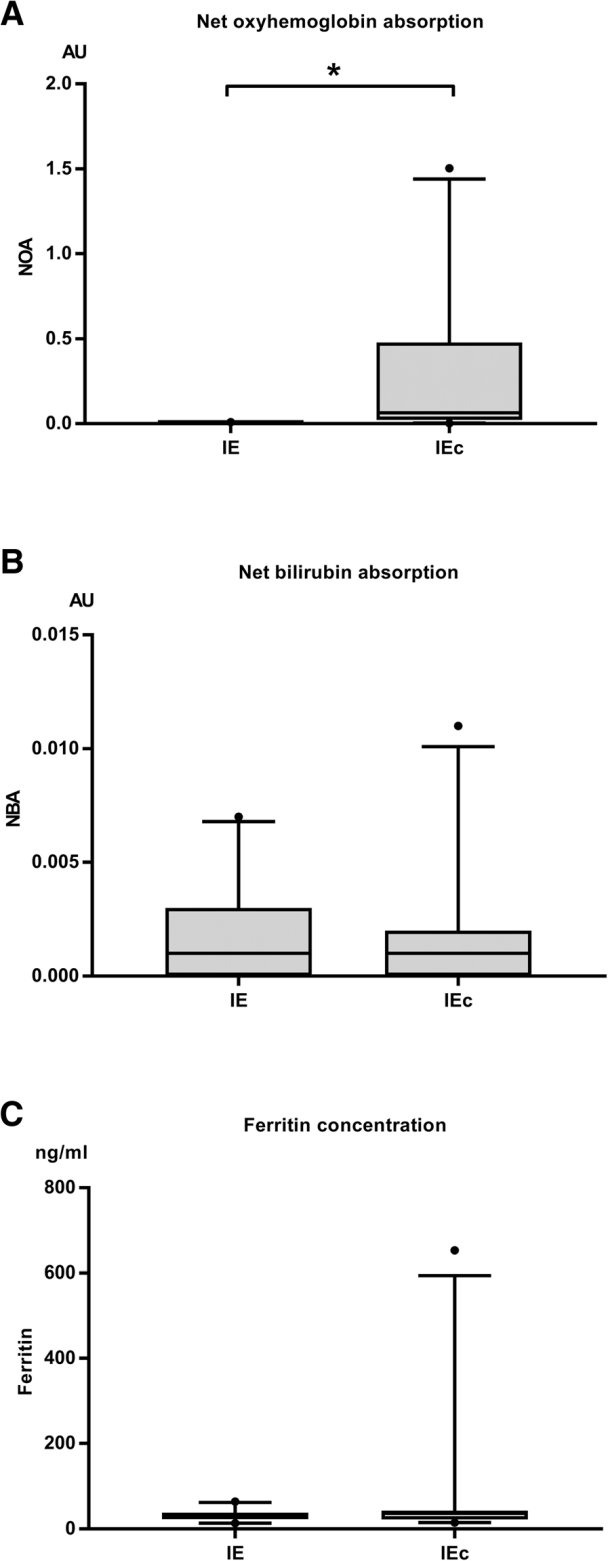
Comparison of (**a**) net oxyhemoglobin absorbance (NOA) (**b**) net bilirubin absorbance (NBA), and (**c**) ferritin concentration in the cerebrospinal fluid (CSF) of dogs with idiopathic epilepsy either non-contaminated (IE) or artificially blood contaminated (IEc). The box represents the 25th, 50th, and 75th percentile of the distribution; the whiskers approximate the 5th–95th percentile; * indicates values of *P* < 0.05

Comparing the IE and IEc group, NOA was significantly higher in the IEc group (*P* < 0.001), and NBA and ferritin concentration were not different between both groups (*P* = 0.272, *P* = 0.546, respectively). (Fig. 1a-c).

**Fig. 2:**
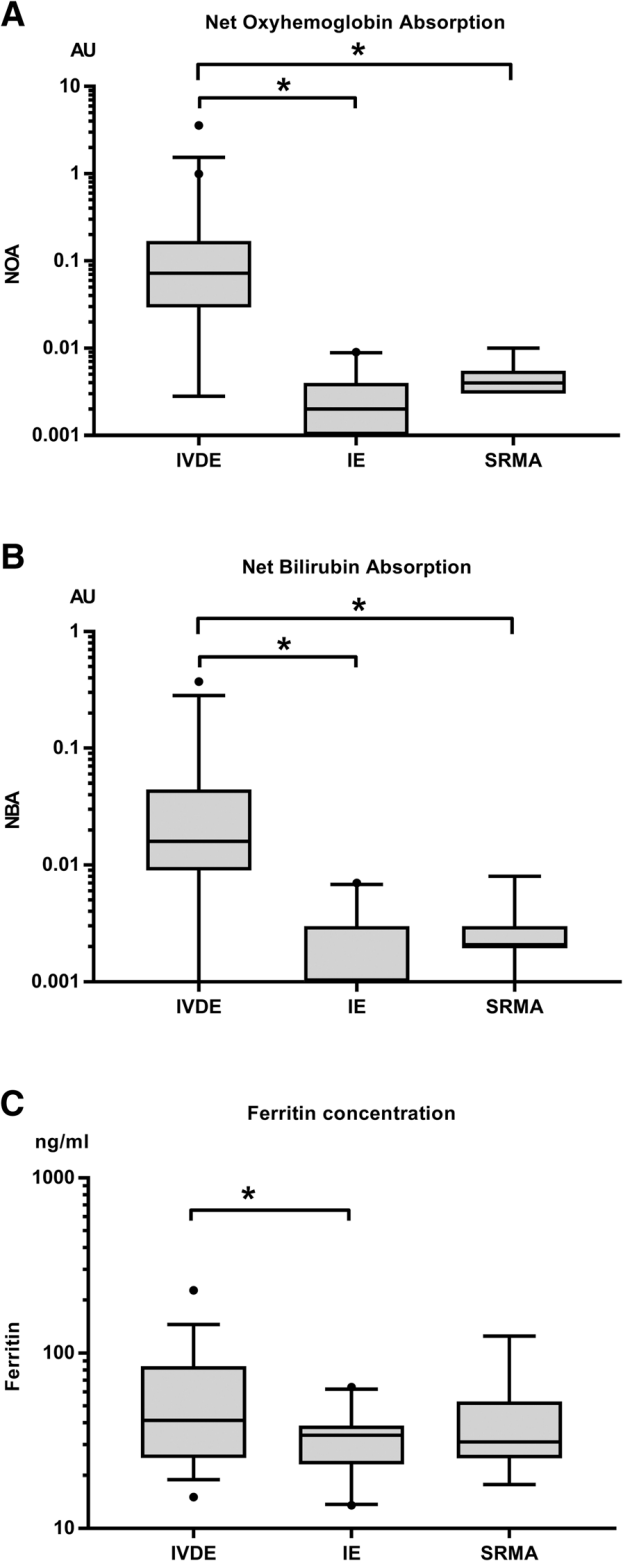
Comparison of (**a**) net oxyhemoglobin absorbance (NOA), (**b**) net bilirubin absorbance (NBA), and (**c**) ferritin concentration in the cerebrospinal fluid (CSF) of dogs with intervertebral disc extrusion (IVDE) to dogs with idiopathic epilepsy (IE) and dogs with steroid responsive meningitis arteritis (SRMA). The box represents the 25th, 50th, and 75th percentile of the distribution; the whiskers approximate the 5th–95th percentile; * indicates values of *P* < 0.05

The NOA and NBA were significantly higher in the CSF of dogs with IVD extrusion compared to dogs with IE (both *P* < 0.001) and SRMA (both *P* < 0.001). (Fig. 2a, b).

The CSF ferritin concentration was significantly higher in dogs with IVD extrusion compared to dogs with IE (*P* = 0.034), but not to dogs with SRMA (*P* = 0.526). (Fig. 2c).

Using the classification according to Cruickshank et al. [27] (Fig. 4), dogs with IVD extrusion showed significantly more often hemorrhage in the subarachnoidal space than dogs with IE and SRMA (both *P* < 0.001). Comparing the non-contaminated and contaminated CSF of the IE dogs according to this classification, the IEc showed significantly more often hemorrhage in the subarachnoidal space than the IE (*P* = 0.002).

In dogs with IVD extrusion, there was no association between NOA, NBA, or ferritin concentration and the initial neurological grade (*P* = 0.104, *P* = 0.081, *P* = 0.704, respectively), and duration of clinical signs (*P* = 0.411, *P* = 0.218, *P* = 0.663, respectively). There was also no association between NOA or NBA and the outcome (*P* = 0.967, *P* = 0.310, respectively), but dogs with a higher ferritin concentration in the CSF had a better outcome (*P* = 0.018).

Using the classification according to Cruickshank et al. there was no association to the outcome (*P* = 0.733).

In dogs with evidence of hemorrhage on MRI, there was a significantly higher NOA and NBA (*P* = 0.016, *P* = 0.009, respectively), but no association with ferritin (*P* = 0.628).

## Discussion

We have shown before that hemorrhage in SCI is associated with the severity of white and gray matter damage and the longitudinal extension of myelomalacia [21]. In addition to the mechanical effects of hemorrhagic cord necrosis [21], it has been a long held view that disruption of the blood-spinal cord barrier with hemorrhage following SCI leads to exposure of the myelon to destructive effects of, among others, cytotoxic neurotransmitters [35], cytokines [36], vasoactive peptides [37], oxygen free radicals [38], endothelin-1 [26, 39, 40] and heme-oxygenase-1 [41]. We hypothesized that detection of blood degradation products in the CSF could be a promising prognostic indicator [21]. A major challenge is to distinguish intra vitam from iatrogenic hemorrhage resulting from CSF puncture. Indeed as reported in people and dogs previously [42, 43], the latter was often suspected since we used lumbar puncture in view of the scope of this study. Counting erythrocytes or measuring hemoglobin cannot be used to distinguish between iatrogenic and true hemorrhage [44]. Detection of erythrophages and siderophages in the CSF can provide information about the age and dynamics of the bleeding, however, studies in the human literature included too few patients to estimate sensitivity for SAH [44, 45]. Spectrophotometry of CSF for bilirubin and oxyhemoglobin is recommended by the British national guidelines in human patients with suspected SAH when computed tomography is negative in order to determine the need for angiography [27]. Spectrophotometry has an estimated sensitivity of 87–100% and specificity of 75–99% for aneurysmal SAH in human patients [31, 46–48]. Bilirubin in CSF may serve as an indicator of central nervous system hemorrhage but only in the absence of severe blood-CSF barrier breakdown, and jaundice [27, 42]. Hence, a decision tree which takes the NOA, NBA, serum bilirubin and CSF protein concentration into consideration is recommended in the British national guidelines to interpret spectrophotometric results from human patients [27]. In the present study, we found that the same decision tree could be used for the assessment of spinal cord hemorrhage in dogs, with the limitation of traumatic puncture.

Following hemorrhage into the subarachnoidal space, rapid lysis of red blood cells occurs within two to four hours as a consequence of the low CSF osmolarity leading to release of oxyhemoglobin, which may be detected between two and 12 h up to one week after the onset of bleeding [44]. However, oxyhemoglobin can also result from in vitro hemolysis after a traumatic puncture [43].

Accordingly, in the present study, NOA was significantly higher following artificial blood contamination of the CSF and subsequent centrifugation within 15 min compared to the same CSF without contamination. Therefore, NOA has a relatively high probability of falsely elevated results especially in lumbar CSF taps due to traumatic puncture in the face of iatrogenic blood contamination, and thus rendering the interpretation of significantly elevated NOA in dogs with IVD extrusion difficult. We concluded that NOA may be of limited value to assess cord hemorrhage.

The liberated oxyhemoglobin is gradually converted into bilirubin and free iron by macrophages and other cells of the leptomeninges [22]. Thus, an elevation of bilirubin cannot be explained by a traumatic puncture, as reflected by the NBA results clearly distinguishing between artificially blood contaminated and non-contaminated CSF of IE dogs. The conversion into bilirubin and iron is detectable about 12 h after onset of hemorrhage [27, 29], increases over approximately one week, and remains detectable for two to four weeks after the hemorrhage in people [22, 31]. In the present study, all CSF taps were performed after more than 12 h and less than 3 weeks after the beginning of clinical symptoms, and we could show a significant elevation of NBA in dogs with SCI compared to controls. However, we could not find a correlation between NBA values and initial neurological grade, duration of clinical signs and outcome in dogs with IVD extrusion which may be due to several variables. The level of bilirubin in the CSF is not only dependent on the quantity of hemorrhage, but also very much on the time point of CSF puncture after SCI, and hence the conversion stage of oxyhemoglobin. The interpretation of NBA values may be further complicated by progression of the IVD disease. For example, in the present study, three of six chronically affected dogs showed an acute worsening of clinical signs possibly by further extrusion of IVD material [49–51] perhaps leading to new hemorrhage. Beside the stage of hemorrhage, the distance of the primary lesion site to the site of CSF tap could also have an influence on the results. In the present study, the CSF tap was performed at L5–6 or L6–7 and the distance to the IVD extrusion site was between zero and seven vertebral bodies.

Ferritin is the main extra-cellular iron transporter, and located in every organ. After hemorrhagic events, it is synthesized by several cell types among others macrophages in order to bind free iron, and to prevent the occurrence of free radicals that damage tissues [52]. The free iron gets detoxified by ferritin which reaches a maximum peak after about eight to 11 days following hemorrhage [23, 24]. The ferritin concentration was significantly higher in the CSF of dogs with IVD extrusion compared to dogs with IE, but not compared to dogs with SRMA. The latter is consistent with previous observations according to which ferritin concentration in the CSF is also increased in inflammation of the central nervous system, especially in pyogenic inflammation [52].

Interestingly, dogs who recovered ambulation had a significantly higher ferritin concentration than dogs with a poor outcome. However, in addition to hemorrhage as a source of iron, the ferritin concentration could also reflect the inflammatory response following SCI, which is extremely complex and has both beneficial and deleterious effect [53–56]. In the spinal cord, ferritin is a marker of microglia, which is important for reestablishing tissue homeostasis after SCI [55, 57–59]. However, the interpretation of ferritin levels in canine CSF was also complicated by its wide concentration range in the control dogs. It normally does not cross the blood brain barrier due to its relative high molecular weight of 450 kD [60], and no correlation between CSF and serum ferritin concentration has been found in people. The upper normal range of ferritin in human CSF is 12 ng/ml. The level of ferritin in the CSF of control dogs with IE in the present study was between 13 and 64 ng/ml. However, to evaluate the normal range of ferritin in the CSF of dogs, a larger case number of completely healthy dogs should be examined.

MRI is the method of choice to evaluate the spinal canal for the presence of hemorrhage until now. However, to distinguish between extradural and subdural hemorrhage, and especially its quantification remains difficult. The amount of blood degradation products in the CSF correlated with the evidence of hemorrhage on MRI in the present study.

Limitations of the present pilot study are the very low number of paraplegic dogs with loss of nociception and the short follow up time of dogs with a poor outcome probably leading to biased statistical results. Additionally the variation in clinical duration (acute vs. acute on chronic vs. chronic) may have affected the measurements. Larger case numbers are needed. Also intraparenchymal hemorrhage is most likely missed with measurements of blood degradation products in the CSF.

## Conclusion

The results of this study show that detection of blood degradation products such as oxyhemoglobin, bilirubin and ferritin in lumbar CSF to demonstrate hemorrhage in the subarachnoidal space of dogs following IVD extrusion is feasible, and correlates with MRI findings. However, while significant differences were found between dogs with IVD disease and controls, correlations of NOA and NBA to the initial neurological grade and the outcome in individual animals could not be found. NOA is not recommended after a lumbar CSF puncture due to the high risk of iatrogenic blood contamination. Paradoxically, dogs who regained ambulation had significantly higher CSF ferritin concentration.

Since the generation of blood degradation products in the CSF is a highly dynamic process bilirubin and ferritin values depend very strongly on the duration of clinical signs. Larger numbers of animals for each defined point in time following initial SCI are necessary to evaluate the usefulness of bilirubin and ferritin as prognostic indicators.

## Methods

### Case selection

Examination of the CSF for blood degradation products was performed prospectively in 39 dogs with thoracolumbar IVD extrusion which were presented between September 2013 and October 2015 at the veterinary teaching hospital of the University of Bern. Inclusion criteria were diagnosis of surgically or histopathologically confirmed thoracolumbar IVD extrusion, well-documented clinical records (breed, age, gender, duration of clinical signs, neurological grade, MRI findings, outcome), and sufficient quantity of CSF (0.5 ml) for measurements. Additionally, CSF of 21 dogs with idiopathic epilepsy (IE) and 9 dogs with steroid responsive meningitis arteritis (SRMA) served as controls.

### Clinical data

Breed, age and gender of all dogs were recorded, and a complete neurological examination was performed at the time of presentation. In dogs with thoracolumbar IVD extrusion, duration and severity of clinical signs, site of IVD extrusion, and outcome were noted.

The initial neurological condition was graded on a I to V scale as published before [3]: Grade I, spinal hyperesthesia only; Grade II, ambulatory paraparesis, ataxia, and proprioceptive deficits; Grade III, non-ambulatory paraparesis; Grade IV, paraplegia with present nociception; and Grade V, paraplegia with loss of nociception. In case of grade differences between left and right limbs, the more severe grade was assigned.

Duration of neurologic signs was defined as the time between the first observed clinical signs and collection of CSF, and was grouped as follows: CSF collection within the first 48 h (acute), within 2–7 days (subacute), or after more than 7 days (chronic) according to previous studies [30].

In all included dogs with thoracolumbar IVD extrusion, the diagnosis was provided by MRI and confirmed during standard hemilaminectomy.

The neurological condition was reevaluated at time of discharge. The outcome was determined as follows: grade 0 - lack of improvement or euthanasia; grade 1 - improvement of the neurological status by at least one grade, but not able to walk without support; grade 2 - recovery to ambulation [20].

In control dogs, the diagnosis of IE or SRMA was based on signalement, history, clinical signs and specific diagnostic testing. Dogs with IE displayed no interictal neurological deficits, metabolic causes were excluded, and MRI of the head and CSF tap were unremarkable. Dogs with SRMA revealed suspicious clinical signs (neck pain, pyrexia), a CSF tap with neutrophilic pleocytosis and without detectable pathologic organisms on cytology, an increased immunoglobulin A level in serum and CSF, and a resolution of clinical signs after treatment with corticosteroids.

### Magnetic resonance imaging

MRI examinations were performed in all dogs using a 1.0-T magnet 1. The MRI protocol usually included a transverse and sagittal T2-weighted fast spin-echo sequence, a sagittal fluid-attenuating inversion recovery sequence (FLAIR), a dorsal short tau inversion recovery sequence (STIR), dorsal and transverse T1-weighted sequences, and a transverse T2*-weighted sequence. Board-certified radiologists qualitatively evaluated the MRI images and judged hemorrhage in the spinal canal (epidural, subdural) as present or not present.

### Serum bilirubin levels and cerebrospinal fluid analysis

A blood sample of all dogs was taken by venipuncture at the time of CSF collection, and serum bilirubin levels were determined [31].

For the measurements of blood degradation products and ferritin, a minimum of 0.5 ml CSF was needed. The CSF tap was performed under general anesthesia at the cisterna magna in dogs with SRMA, and subsequently following MRI in dogs with IE. A lumbar tap (L5–6 or L6–7) was performed following MRI in dogs with thoracolumbar IVD extrusion.

In dogs with IVD extrusion, 0.5 ml of the CSF was processed immediately for measurements of blood degradation products (see below), and if provided that a sufficient quantity of CSF remained, routine CSF analysis was performed including white blood cell (WBC) count, total protein, pandy testing, and in cases with a WBC count > 5 / microliter cytology following cytospin preparation.

To evaluate the potential effect of blood contamination during CSF puncture on spectrophotometric and ferritin measurements, the CSF samples from the 21 dogs with IE were divided in two parts (each at least 0.5 ml), and one part was artificially contaminated with a drop of blood (IEc).

**Fig. 3:**
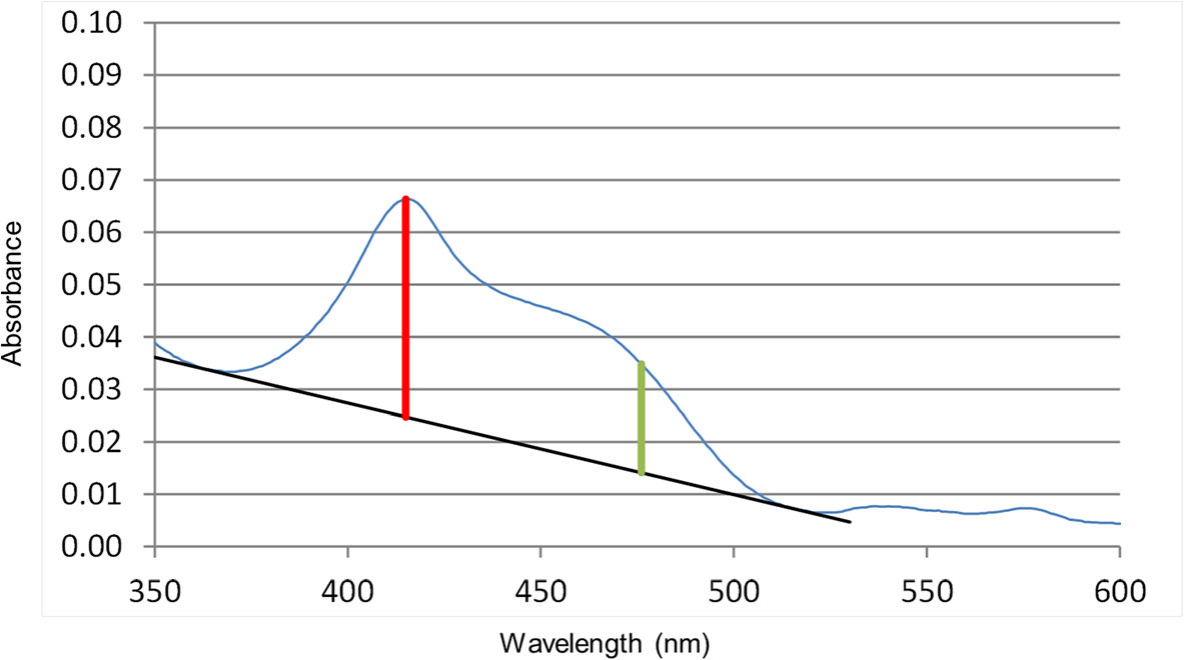
Representative spectrophotometric scan (blue curve). A predicted baseline (black), which forms a tangent to the scan between 350 and 400 nm and between 430 and 530 nm was drawn. The vertical reference line at 476 nm represents the net bilirubin absorbance (green), and the vertical reference line at any peak between 410 and 418 nm the net oxyhemoglobin absorbance (red)

All CSF samples, were protected from light immediately after collection, centrifuged (10 min, 2360 g, 4 °C) within 15 min, and the supernatant stored in Eppendorf-tubes at − 20 °C until further diagnostics [27, 32, 33]. The samples were analyzed at wave lengths between 350 and 660 nm using a Shimadzu UV-1800 spectrophotometer 2 in the Center of Laboratory Medicine, University Institute of Clinical Chemistry (Inselspital). NOA and NBA were determined in the CSF according the Chalmers’ calculation [34]. A predicted baseline, which forms a tangent to the scan between 350 and 400 nm and again between 430 and 530 nm was drawn. The absorbance above this predicted baseline at 476 nm reflects the NBA. If the baseline forms a tangent to the scan before 476 nm, the measured NBA is by definition zero. The NOA is represented by any oxyhemoglobin peak above this predicted baseline (Fig. 3) [27].

**Fig. 4:**
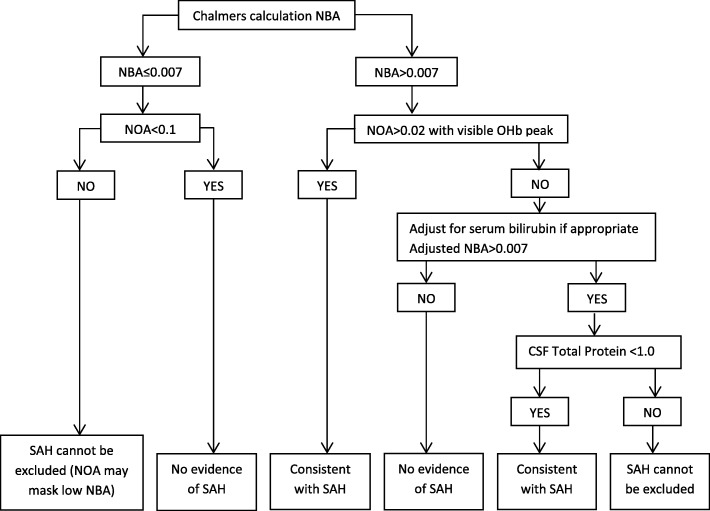
Detection of hemorrhage in the cerebrospinal fluid (CSF) using net bilirubin absorbance (NBA) by continuing a modified decision tree [27] (NOA, net oxyhemoglobin absorbance)

Additionally, the measurements were interpreted in respect to the serum bilirubin and CSF total protein level by following a recommended decision tree [27]: (1) no evidence of SAH, or (2) consistent with SAH, or (3) SAH cannot be excluded (Fig. 4).

Ferritin was determined quantitatively in the CSF using a commercially available canine ferritin ELISA kit 3. Every sample was measured in duplicates. Subsequently; the mean was calculated and used for statistical evaluation.

### Statistical analysis

Dogs were assigned to one of the following four groups: (1) dogs with thoracolumbar IVD extrusion, (2) dogs with IE, (3) dogs with IE and CSF artificially blood contaminated (IEc), and (4) dogs with SRMA.

For statistical analysis, the neurological grade of dogs with IVD extrusion at the initial presentation was classed together to ambulatory paraparesis (grade I, II), non-ambulatory paraparesis (grade III), and paraplegia (grade IV, V). Due to small case numbers, the outcome grades 0 and 1 were merged.

Statistical evaluation was performed using the software package NCSS 9 (http://www.ncss.com). The threshold value for statistical significance was set at *P <* 0.05.

Since data were not normally distributed non-parametric tests were used. The Kruskal-Wallis test was used to evaluate the equality of variance for NOA, NBA, ferritin, and the classification according to Cruickshank et al. [27]. Multiple comparisons between dogs with IVD extrusion and the control dogs with IE or SRMA were performed using the Kruskal-Wallis test and the Wilcoxon two-sample test, respectively. The influence of iatrogenic blood contamination was evaluated by comparing NOA, NBA, ferritin concentration, and the classification according to Cruickshank et al. [27] between the IE and IEc group using the Wilcoxon two-sample test.

In dogs with thoracolumbar IVD extrusion, associations between NOA, NBA, ferritin, and the classification according to Cruickshank et al. [27] in the CSF and (1) the initial neurological grade, (2) the duration of clinical signs, (3) the outcome, and (4) MRI findings were statistically evaluated using the Kruskal-Wallis test and the Wilcoxon two-sample test, respectively. Results are given as median and range (5th–95th percentile).

## Abbreviations

CSF: Cerebrospinal fluid
IE: Idiopathic epilepsy
IEc: Idiopathic epilepsy contaminated
IVD: Intervertebral disk
MRI: Magnetic resonance imaging
NBA: Net bilirubin absorption
NOA: Net oxyhemoglobin absorption
SAH: Subarachnoidal hemorrhage
SCI: Spinal cord injury
SRMA: Steroid responsive meningitis arteritis
WBC: White blood cell count

## Footnotes

1 Panorama HFO, Philips Medical Systems

2 UV/VIS spectrophotometer Shimadzu UV-1800, Shimadzu, Kyoto, Japan

3 Canine ferritin ELISA kit, BlueGene Biotech, Putuo District, Shanghai, China

## References

[1] Scott HW (1997). Hemilaminectomy for the treatment of thoracolumbar disc disease in the dog: a follow-up study of 40 cases. J Small Anim Pract.

[2] Scott HW, McKee WM (1999). Laminectomy for 34 dogs with thoracolumbar intervertebral disc disease and loss of deep pain perception. J Small Anim Pract.

[3] Henke D., Vandevelde M., Doherr M.G., Stöckli M., Forterre F. (2013). Correlations between severity of clinical signs and histopathological changes in 60 dogs with spinal cord injury associated with acute thoracolumbar intervertebral disc disease. The Veterinary Journal.

[4] Duval J, Dewey C, Roberts R, Aron D (1996). Spinal cord swelling as a myelographic indicator of prognosis: a retrospective study in dogs with intervertebral disc disease and loss of deep pain perception. Veterinary surgery : VS.

[5] Olby N, Levine J, Harris T, Munana K, Skeen T, Sharp N (2003). Long-term functional outcome of dogs with severe injuries of the thoracolumbar spinal cord: 87 cases (1996-2001). J Am Vet Med Assoc.

[6] Laitinen OM, Puerto DA (2005). Surgical decompression in dogs with thoracolumbar intervertebral disc disease and loss of deep pain perception: a retrospective study of 46 cases. Acta Vet Scand.

[7] Ito D, Matsunaga S, Jeffery ND, Sasaki N, Nishimura R, Mochizuki M, Kasahara M, Fujiwara R, Ogawa H (2005). Prognostic value of magnetic resonance imaging in dogs with paraplegia caused by thoracolumbar intervertebral disk extrusion: 77 cases (2000-2003). J Am Vet Med Assoc.

[8] Penning V, Platt SR, Dennis R, Cappello R, Adams V (2006). Association of spinal cord compression seen on magnetic resonance imaging with clinical outcome in 67 dogs with thoracolumbar intervertebral disc extrusion. J Small Anim Pract.

[9] De Risio L, Adams V, Dennis R, McConnell FJ (2009). Association of clinical and magnetic resonance imaging findings with outcome in dogs with presumptive acute noncompressive nucleus pulposus extrusion: 42 cases (2000-2007). J Am Vet Med Assoc.

[10] Srugo I, Aroch I, Christopher MM, Chai O, Goralnik L, Bdolah-Abram T, Shamir MH (2011). Association of cerebrospinal fluid analysis findings with clinical signs and outcome in acute nonambulatory thoracolumbar disc disease in dogs. J Vet Intern Med.

[11] Levine GJ, Cook JR, Kerwin SC, Mankin J, Griffin JF, Fosgate GT, Levine JM (2014). Relationships between cerebrospinal fluid characteristics, injury severity, and functional outcome in dogs with and without intervertebral disk herniation. Vet Clin Pathol.

[12] Chamisha Y, Aroch I, Kuzi S, Srugo I, Bdolah-Abram T, Chai O, Christopher MM, Merbl Y, Rothwell K, Shamir MH (2015). The prognostic value of cerebrospinal fluid characteristics in dogs without deep pain perception due to thoracolumbar disc herniation. Res Vet Sci.

[13] Nagano S, Kim SH, Tokunaga S, Arai K, Fujiki M, Misumi K (2011). Matrix metalloprotease-9 activity in the cerebrospinal fluid and spinal injury severity in dogs with intervertebral disc herniation. Res Vet Sci.

[14] Anderson KM, Welsh CJ, Young C, Levine GJ, Kerwin SC, Boudreau CE, Reyes I, Mondragon A, Griffin JF, Cohen ND (2015). Acute phase proteins in cerebrospinal fluid from dogs with naturally-occurring spinal cord injury. J Neurotrauma.

[15] Roerig A, Carlson R, Tipold A, Stein VM (2013). Cerebrospinal fluid tau protein as a biomarker for severity of spinal cord injury in dogs with intervertebral disc herniation. Vet J.

[16] Levine GJ, Levine JM, Witsberger TH, Kerwin SC, Russell KE, Suchodolski J, Steiner J, Fosgate GT (2010). Cerebrospinal fluid myelin basic protein as a prognostic biomarker in dogs with thoracolumbar intervertebral disk herniation. J Vet Intern Med.

[17] Witsberger TH, Levine JM, Fosgate GT, Slater MR, Kerwin SC, Russell KE, Levine GJ (2012). Associations between cerebrospinal fluid biomarkers and long-term neurologic outcome in dogs with acute intervertebral disk herniation. J Am Vet Med Assoc.

[18] Olby N (2010). The pathogenesis and treatment of acute spinal cord injuries in dogs. Vet Clin North Am Small Anim Pract.

[19] Dumont RJ, Okonkwo DO, Verma S, Hurlbert RJ, Boulos PT, Ellegala DB, Dumont AS (2001). Acute spinal cord injury, part I: pathophysiologic mechanisms. Clin Neuropharmacol.

[20] Fadda A, Oevermann A, Vandevelde M, Doherr MG, Forterre F, Henke D (2013). Clinical and pathological analysis of epidural inflammation in intervertebral disk extrusion in dogs. J Vet Intern Med.

[21] Henke D, Gorgas D, Doherr MG, Howard J, Forterre F, Vandevelde M (2016). Longitudinal extension of myelomalacia by intramedullary and subdural hemorrhage in a canine model of spinal cord injury. Spine J.

[22] Barrows LJ, Hunter FT, Banker BQ (1955). The nature and clinical significance of pigments in the cerebrospinal fluid. Brain.

[23] Suzuki H, Muramatsu M, Tanaka K, Fujiwara H, Kojima T, Taki W (2006). Cerebrospinal fluid ferritin in chronic hydrocephalus after aneurysmal subarachnoid hemorrhage. J Neurol.

[24] Petzold A, Worthington V, Appleby I, Kerr ME, Kitchen N, Smith M (2011). Cerebrospinal fluid ferritin level, a sensitive diagnostic test in late-presenting subarachnoid hemorrhage. Journal of stroke and cerebrovascular diseases : the official journal of National Stroke Association.

[25] Sinescu C, Popa F, Grigorean VT, Onose G, Sandu AM, Popescu M, Burnei G, Strambu V, Popa C (2010). Molecular basis of vascular events following spinal cord injury. J Med Life.

[26] Mayer D, Oevermann A, Seuberlich T, Vandevelde M, Casanova-Nakayama A, Selimovic-Hamza S, Forterre F, Henke D (2016). Endothelin-1 immunoreactivity and its association with intramedullary hemorrhage and Myelomalacia in naturally occurring disk extrusion in dogs. J Vet Intern Med.

[27] Cruickshank A, Auld P, Beetham R, Burrows G, Egner W, Holbrook I, Keir G, Lewis E, Patel D, Watson I (2008). Revised national guidelines for analysis of cerebrospinal fluid for bilirubin in suspected subarachnoid haemorrhage. Ann Clin Biochem.

[28] Marlet JM, Barreto Fonseca JP (1982). Experimental determination of time of intracranial hemorrhage by spectrophotometric analysis of cerebrospinal fluid. J Forensic Sci.

[29] Morgan CJ, Pyne-Geithman GJ, Jauch EC, Shukla R, Wagner KR, Clark JF, Zuccarello M (2004). Bilirubin as a cerebrospinal fluid marker of sentinel subarachnoid hemorrhage: a preliminary report in pigs. J Neurosurg.

[30] Karli P, Martle V, Bossens K, Summerfield A, Doherr MG, Turner P, Vandevelde M, Forterre F, Henke D (2014). Dominance of chemokine ligand 2 and matrix metalloproteinase-2 and -9 and suppression of pro-inflammatory cytokines in the epidural compartment after intervertebral disc extrusion in a canine model. Spine J.

[31] Alons IM, Verheul RJ, Ponjee GA, Jellema K (2013). Optimizing blood pigment analysis in cerebrospinal fluid for the diagnosis of subarachnoid haemorrhage--a practical approach. Eur J Neurol.

[32] Wahlgren NG, Bergstrom K (1983). Determination of haem derivatives in the cerebrospinal fluid--a semi-quantitative method. J Neurol Neurosurg Psychiatry.

[33] Thoresen SI, Tverdal A, Havre G, Morberg H (1995). Effects of storage time and freezing temperature on clinical chemical parameters from canine serum and heparinized plasma. Vet Clin Pathol.

[34] Chalmers AH (2001). Cerebrospinal fluid xanthochromia testing simplified. Clin Chem.

[35] Schlosshauer B (1993). The blood-brain barrier: morphology, molecules, and neurothelin. Bioessays.

[36] Knerlich-Lukoschus F, Krossa S, Krause J, Mehdorn HM, Scheidig A, Held-Feindt J (2015). Impact of chemokines on the properties of spinal cord-derived neural progenitor cells in a rat spinal cord lesion model. J Neurosci Res.

[37] Bartholdi D, Schwab ME (1997). Expression of pro-inflammatory cytokine and chemokine mRNA upon experimental spinal cord injury in mouse: an in situ hybridization study. Eur J Neurosci.

[38] Lewen A, Matz P, Chan PH (2000). Free radical pathways in CNS injury. J Neurotrauma.

[39] Westmark R, Noble LJ, Fukuda K, Aihara N, McKenzie AL (1995). Intrathecal administration of endothelin-1 in the rat: impact on spinal cord blood flow and the blood-spinal cord barrier. Neurosci Lett.

[40] Mautes AE, Weinzierl MR, Donovan F, Noble LJ (2000). Vascular events after spinal cord injury: contribution to secondary pathogenesis. Phys Ther.

[41] Mautes AE, Kim DH, Sharp FR, Panter S, Sato M, Maida N, Bergeron M, Guenther K, Noble LJ (1998). Induction of heme oxygenase-1 (HO-1) in the contused spinal cord of the rat. Brain Res.

[42] Wick M, Fink W, Pfister W, Einhaupl K, Huber M, Fateh-Moghadam A (1988). Ferritin in cerebrospinal fluid differentiation between central nervous system haemorrhage and traumatic spinal puncture. J Clin Pathol.

[43] Beetham R (2009). CSF spectrophotometry for bilirubin--why and how?. Scand J Clin Lab Invest.

[44] Nagy K, Skagervik I, Tumani H, Petzold A, Wick M, Kuhn HJ, Uhr M, Regeniter A, Brettschneider J, Otto M (2013). Cerebrospinal fluid analyses for the diagnosis of subarachnoid haemorrhage and experience from a Swedish study. What method is preferable when diagnosing a subarachnoid haemorrhage?. Clin Chem Lab Med.

[45] Page KB, Howell SJ, Smith CM, Dabbs DJ, Malia RG, Porter NR, Thickett KJ, Wilkinson GM (1994). Bilirubin, ferritin, D-dimers and erythrophages in the cerebrospinal fluid of patients with suspected subarachnoid haemorrhage but negative computed tomography scans. J Clin Pathol.

[46] Chao CY, Florkowski CM, Fink JN, Southby SJ, George PM (2007). Prospective validation of cerebrospinal fluid bilirubin in suspected subarachnoid haemorrhage. Ann Clin Biochem.

[47] Wood MJ, Dimeski G, Nowitzke AM (2005). CSF spectrophotometry in the diagnosis and exclusion of spontaneous subarachnoid haemorrhage. J Clin Neurosci.

[48] Chu K, Hann A, Greenslade J, Williams J, Brown A (2014). Spectrophotometry or visual inspection to most reliably detect xanthochromia in subarachnoid hemorrhage: systematic review. Ann Emerg Med.

[49] Brisson BA, Moffatt SL, Swayne SL, Parent JM (2004). Recurrence of thoracolumbar intervertebral disk extrusion in chondrodystrophic dogs after surgical decompression with or without prophylactic fenestration: 265 cases (1995-1999). J Am Vet Med Assoc.

[50] Forterre F, Gorgas D, Dickomeit M, Jaggy A, Lang J, Spreng D (2010). Incidence of spinal compressive lesions in chondrodystrophic dogs with abnormal recovery after hemilaminectomy for treatment of thoracolumbar disc disease: a prospective magnetic resonance imaging study. Veterinary surgery : VS.

[51] Roach WJ, Thomas M, Weh JM, Bleedorn J, Wells K (2012). Residual herniated disc material following hemilaminectomy in chondrodystrophic dogs with thoracolumbar intervertebral disc disease. Vet Comp Orthop Traumatol.

[52] Kolodziej MA, Proemmel P, Quint K, Strik HM (2014). Cerebrospinal fluid ferritin--unspecific and unsuitable for disease monitoring. Neurol Neurochir Pol.

[53] Shechter R, London A, Varol C, Raposo C, Cusimano M, Yovel G, Rolls A, Mack M, Pluchino S, Martino G (2009). Infiltrating blood-derived macrophages are vital cells playing an anti-inflammatory role in recovery from spinal cord injury in mice. PLoS Med.

[54] Kigerl KA, Gensel JC, Ankeny DP, Alexander JK, Donnelly DJ, Popovich PG (2009). Identification of two distinct macrophage subsets with divergent effects causing either neurotoxicity or regeneration in the injured mouse spinal cord. J Neurosci.

[55] Alexander JK, Popovich PG (2009). Neuroinflammation in spinal cord injury: therapeutic targets for neuroprotection and regeneration. Prog Brain Res.

[56] Donnelly DJ, Popovich PG (2008). Inflammation and its role in neuroprotection, axonal regeneration and functional recovery after spinal cord injury. Exp Neurol.

[57] Faulkner JR, Herrmann JE, Woo MJ, Tansey KE, Doan NB, Sofroniew MV (2004). Reactive astrocytes protect tissue and preserve function after spinal cord injury. J Neurosci.

[58] Pender MP, Rist MJ (2001). Apoptosis of inflammatory cells in immune control of the nervous system: role of glia. Glia.

[59] Wojtera M, Sobow T, Kloszewska I, Liberski PP, Brown DR, Sikorska B (2012). Expression of immunohistochemical markers on microglia in Creutzfeldt-Jakob disease and Alzheimer's disease: morphometric study and review of the literature. Folia Neuropathol.

[60] Milman N, Graudal NA, Olsen TS, Wandall JH, Pedersen NS (1993). Cerebrospinal fluid ferritin in patients with meningitis and cerebral infarction or bleeding. Dan Med Bull.

